# Genome-wide association mapping of lentil (*Lens culinaris* Medikus) prebiotic carbohydrates toward improved human health and crop stress tolerance

**DOI:** 10.1038/s41598-021-93475-3

**Published:** 2021-07-06

**Authors:** Nathan Johnson, J. Lucas Boatwright, William Bridges, Pushparajah Thavarajah, Shiv Kumar, Emerson Shipe, Dil Thavarajah

**Affiliations:** 1 Plant and Environmental Sciences, 113 Biosystems Research Complex, Clemson University, Clemson, SC 29634 USA; 2 Advanced Plant Technology, Clemson University, Clemson, SC 29634 USA; 3 Biodiversity and Crop Improvement Program, International Centre for Agricultural Research in the Dry Areas (ICARDA), Rabat-Institute, P.O. Box 6299, Rabat, Morocco

**Keywords:** Carbohydrates, Agricultural genetics, Genotype, Plant breeding, Plant genetics

## Abstract

Lentil, a cool-season food legume, is rich in protein and micronutrients with a range of prebiotic carbohydrates, such as raffinose-family oligosaccharides (RFOs), fructooligosaccharides (FOSs), sugar alcohols (SAs), and resistant starch (RS), which contribute to lentil's health benefits. Beneficial microorganisms ferment prebiotic carbohydrates in the colon, which impart health benefits to the consumer. In addition, these carbohydrates are vital to lentil plant health associated with carbon transport, storage, and abiotic stress tolerance. Thus, lentil prebiotic carbohydrates are a potential nutritional breeding target for increasing crop resilience to climate change with increased global nutritional security. This study phenotyped a total of 143 accessions for prebiotic carbohydrates. A genome-wide association study (GWAS) was then performed to identify associated variants and neighboring candidate genes. All carbohydrates analyzed had broad-sense heritability estimates (*H*^2^) ranging from 0.22 to 0.44, comparable to those reported in the literature. Concentration ranges corresponded to percent recommended daily allowances of 2–9% SAs, 7–31% RFOs, 51–111% RS, and 57–116% total prebiotic carbohydrates. Significant SNPs and associated genes were identified for numerous traits, including a galactosyltransferase (*Lcu.2RBY.1g019390*) known to aid in RFO synthesis. Further studies in multiple field locations are necessary. Yet, these findings suggest the potential for molecular-assisted breeding for prebiotic carbohydrates in lentil to support human health and crop resilience to increase global food security.

## Introduction

The World Health Organization estimates that non-communicable diseases (NCDs), such as cardiovascular disease and diabetes, cause 71% of global deaths^1^. The United Nations Sustainable Goals by 2030 include the reduction of NCD mortality by one-third as a primary health goal^1^. NCD risk factors are diverse; however, some, such as obesity, overweight, and malnutrition, clearly have a dietary link. Consequently, food security and consumer acceptance of nutritious foods are vital to lowering NCD risk. Compounding the problem is the threat of climate change to global food security^2^. Anticipated increases in temperature and drought will have harmful effects on crop yields and the people dependent upon them. Thus, ensuring the production of nutritionally dense staple food crops, such as pulses, is essential to address these global food security challenges. Amid the complexity of these issues, we put forward lentil (*Lens culinaris* Medikus), a staple food crop rich in prebiotic carbohydrates, as one piece in the broader solution. Lentil prebiotic carbohydrates are an ideal target for genomic-assisted breeding approaches to combat NCD and ensure global food security.

Lentil is a nutritionally dense cool-season pulse crop with notable concentrations of protein (20–30%), low-digestible carbohydrates (20%), fat (1%), iron (Fe), zinc (Zn), and a range of vitamins^3^. A study in rats shows a lentil diet can significantly lower mean body weight, percent body fat, and blood plasma triglyceride levels and increase lean body mass than control or corn diet^4^. Lentil's health benefits are in part due to its high concentrations of prebiotic or low-digestible carbohydrates, including raffinose-family oligosaccharides (RFOs; 4071 mg/100 g), sugar alcohols (SAs; 1423 mg/100 g), fructooligosaccharides (FOSs; 62 mg/100 g), and resistant starch (RS; 7500 mg/100 g)^5^. A prebiotic is *"a substrate that is selectively utilized by host microorganisms conferring a health benefit"*^6^. When consumed, prebiotics pass through the upper gastrointestinal tract and are fermented by beneficial microorganisms in the colon, which benefits their human host. The human gastrointestinal tract is lined with trillions of microorganisms, composing the microbiome^7^. These microbes are vital to colon health and function, aiding in immune system stimulation, nutrient breakdown and absorption, and bowel motility^8^. Adverse microbiome compositions have been associated with various ailments, such as obesity, diabetes, infection, and colon cancer^9^. Modulation of the microbiome, primarily through prebiotic consumption, can improve health outcomes. For example, a prebiotic-rich diet restored the microbiome composition and plasma biomarkers of malnourished Bangladeshi children to levels similar to healthy children^10^.

Lentil prebiotic carbohydrates also serve a vital role in plant health. Lentil accumulates RFOs in its seeds at high concentrations. Although few studies have been done on lentil RFOs, soybean seedlings have been shown to use this carbon store for energy; however, RFOs do not appear necessary for successful germination^11^. Abiotic stress studies in *Arabidopsis thaliana* show upregulation of RFOs under drought, salinity, cold, and heat stress^12,13^. Further, a transgenic *A. thaliana* line overexpressing three genes essential in RFO synthesis demonstrated increased drought, salinity, and cold tolerance^12^. Similar results are reported for SAs^14^. These carbohydrates function as osmoregulants, cell signals, free radical scavengers, and compatible solutes for enzyme function^15^.

As a staple food crop, lentil may be ideal for marker-assisted breeding efforts to alter prebiotic carbohydrate concentrations to reduce NCDs and advance global food security, now threatened by climate change. However, traditional breeding techniques are particularly challenging for quantitative nutritional traits in mature seeds. Analysis by high-performance anion-exchange chromatography is time-consuming and expensive; therefore, molecular techniques have been explored as a way to significantly accelerate the breeding process^16,17^. Genome-wide association studies (GWAS) can detect quantitative trait loci (QTL) associated with prebiotic carbohydrate concentrations and help identify genetic markers needed for molecular breeding techniques. Lentil is a diploid (2n = 14) with a large ~ 4 Gb genome^18^. This allows for the use of numerous tools developed for diploid crops and simplifies some analysis. However, the large repetitive genome poses some additional challenges, such as generating a reference genome (yet unpublished) and sequencing new lines. One of the advantages of using genotyping-by-sequencing (GBS) methods is eliminating some of this complexity by reducing repetitive DNA sequencing^19^. GWAS using genotyping-by-sequencing (GBS) data have identified markers for *Aphanomyces* root rot resistance^20^ and abiotic stress tolerance^21^ in lentil. However, this is the first comprehensive study to report GWAS findings for prebiotic carbohydrates in lentil. Two lentil mapping populations were obtained from the International Centre for Agricultural Research in the Dry Areas (ICARDA), Rabat Institute, Rabat, Morocco. The heat tolerance population (150 accessions) and the global mapping population (128 accessions) were grown in a completely randomized design with two replicates at the Clemson University Greenhouse Complex, Clemson, SC, USA. The objectives of this study were to (1) identify and quantify prebiotic carbohydrates in a lentil association mapping population grown under greenhouse conditions, (2) identify SNP markers and candidate genes for lentil prebiotic carbohydrates through GWAS, and (3) identify lentil prebiotic carbohydrate breeding targets for human nutrition and climate resilience.

## Results

### Population composition

The two lentil mapping populations were combined for statistical analysis, and an additional 14 lines were added for which data was available. Due to population overlap and poor grain yields, the total number of unique accessions with low-molecular-weight carbohydrate data was 143 with 1–5 replicates per accession. The lentil population included 60 from Asia, 40 from Europe, 16 from Africa, 13 from North America, eight from ICARDA, and six from South America (Table 1).

**Table 1:** *Lens culinaris* ssp. *culinaris* population origin information.

Continent	Country	Accessions
Africa (16)	Algeria (2)	ILL858, ILL4781
	Egypt (2)	ILL702, ILL1046
	Ethiopia (4)	ILL1706, ILL1959, ILL5639, ILL5956
	Libya (1)	ILL4804
	Morocco (2)	ILL6493, ILL7727
	Sudan (2)	ILL1861, ILL5505
	Tunisia (3)	ILL918, ILL1890, ILL6272
Asia (60)	Afghanistan (2)	ILL213, ILL2217
	Armenia (2)	ILL86, ILL619
	Azerbaijan (1)	ILL1671
	Bangladesh (3)	ILL7774, ILL7789, ILL8007
	India (6)	ILL931, ILL3517, ILL4152, ILL4164, ILL4900, ILL5151
	Iran (8)	ILL223, ILL257, ILL769, ILL1013, ILL1097, ILL2406, ILL4791, ILL4886
	Iraq (1)	ILL4899
	Jordan (4)	ILL2150, ILL5261, ILL5384, ILL6925
	Lebanon (3)	ILL191, ILL840, ILL5626
	Nepal (4)	ILL3485, ILL3487, ILL7437, ILL8010
	Pakistan (3)	ILL2297, ILL7650, ILL8114
	Palestine (1)	ILL4606
	Russia (3)	ILL597, ILL4819, ILL4830
	Saudi Arabia (1)	ILL7745
	Syria (6)	ILL158, ILL490, ILL4471, ILL5509, ILL5595, ILL6644
	Tajikistan (2)	ILL598, ILL6126
	Turkey (7)	ILL71, ILL129, ILL550, ILL556, ILL635, ILL2181, ILL6149
	Uzbekistan (1)	ILL4875
	Yemen (2)	ILL950, ILL6281
Europe (40)	Albania (1)	ILL4841
	Belgium (1)	ILL224, ILL6185, ILL7495
	Croatia (1)	ILL4915
	Cyprus (2)	ILL890, ILL5968
	Czech Republic (1)	ILL4409
	France (1)	ILL6528
	Germany (2)	ILL4831, ILL4881
	Greece (4)	ILL304, ILL4857, ILL5519, ILL5533
	Hungary (1)	ILL719
	Italy (4)	ILL343, ILL5416, ILL5418, ILL7084
	North Macedonia (2)	ILL623, ILL624
	Norway (1)	ILL4782
	Poland (2)	ILL705, ILL5424
	Portugal (1)	ILL4956
	Romania (1)	ILL4774
	Serbia and Montenegro (1)	ILL1949
	Spain (4)	ILL4926, ILL5028, ILL5653, Pardina
	Ukraine (3)	ILL82, ILL595, ILL7090
	United Kingdom (3)	ILL348, ILL4345, ILL6415
	Yugoslavia (2)	ILL2230, ILL2231
ICARDA (8)	ICARDA (8)	ILL6994, ILL7012, ILL7978, ILL7979, ILL7981, ILL9888, ILL10053, ILL10281
North America (13)	Canada (4)	ILL4738, Eston, Richlea, Viceroy
	Guatemala (1)	ILL494
	Mexico (3)	ILL502, ILL5553, ILL5645
	United States (5)	ILL4671, Brewer, Crimson, Merrit, Redchief
South America (6)	Argentina (2)	ILL268, ILL4605
	Chile (2)	ILL956, ILL1005
	Columbia (1)	ILL1649
	Uruguay (1)	ILL4778

Numbers in parentheses are accession counts per location.

### Prebiotic carbohydrates

**Figure 1:**
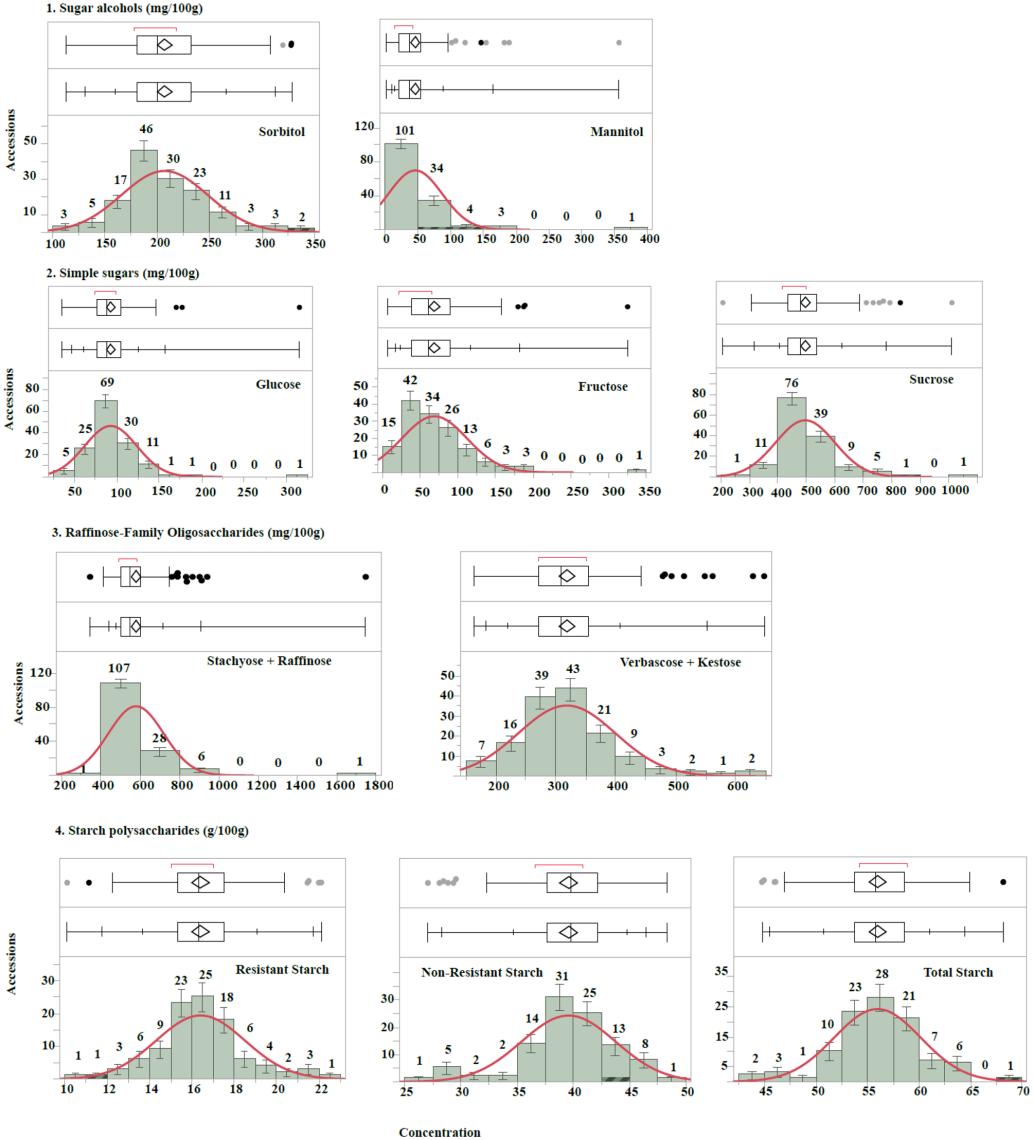
Histograms of accession means with normal curve fits. 1. Sugar alcohols (mg/100 g); Simple sugars (mg/100 g); Raffinose-family oligosaccharides (mg/100 g); starch polysaccharides (g/100 g). The first box plot (Tukey outlier) shows possible outliers as points, while the second box plot (normal quantile) includes all data in estimates. Red normal curves were fitted to the data based on the mean, standard deviation, and sample size.

Low-molecular-weight carbohydrate analysis was conducted on 143 accessions with 1–5 replicates (Table 2). Starch data were only collected from the heat tolerance population and included 102 accessions with 1–2 replicates (Table 2). Mean carbohydrate concentrations (used in the GWAS) were approximately normally distributed, as indicated by the normal red curves fitted to the concentration histograms (Fig. 1). For SAs, sorbitol (sor) had a mean concentration of approximately 4.5 times that of mannitol (man), at 206.8 and 46.8 mg/100 g, respectively. Simple sugars glucose (glu), fructose (fru), and sucrose (suc) had mean concentrations of 93, 69, and 496 mg/100 g, respectively. RFOs stachyose + raffinose (sta + raf) and verbascose + kestose (ver + kes) had mean concentrations of 578 and 318 mg/100 g, respectively (Table 2). Sta + raf and suc had the highest concentrations of all low-molecular-weight carbohydrates measured. Polysaccharides included RS, non-resistant starch (NRS), and total starch (TS) and had mean concentrations of 16.4, 39.6, and 56.0 g/100 g, respectively. All carbohydrates analyzed had modest broad-sense heritability estimates (H^2^) ranging from 0.22 (TS) to 0.45 (man). Concentration ranges corresponding to 2–9%, 7–31%, 51–111%, and 57–116% of the recommended daily allowance (RDA) for SAs, RFOs, RS, and total prebiotic carbohydrates, respectively.

**Table 2:** Carbohydrate analysis with the number of accessions (N), range, overall mean with standard error (SE), and heritability estimates (*H*^2^).

Carbohydrate type	N	Range	Mean ± (SE)	Genotype	*H*^2^	%RDA^a^
**Sugar alcohols (mg/100 g)**						
Sorbitol	143	113–328	207 ± 3	***	0.34	
Mannitol	143	2–357	46 ± 3	***	0.45	
Total sugar alcohols	143	126–609	253 ± 5			2–9
**Simple sugars (mg/100 g)**						
Glucose	143	36–315	93 ± 3	***	0.20	
Fructose	143	7–325	69 ± 4	***	0.23	
Sucrose	143	208–1010	496 ± 9	***	0.34	
Total simple sugars	143	275–1326	658 ± 12			
**Raffinose-family oligosaccharides (mg/100 g)**						
Stachyose + raffinose	143	344–1748	578 ± 12	***	0.41	
Verbascose + kestose	143	164–647	318 ± 7	***	0.29	
Total RFOs	143	508–2167	896 ± 16			7–31
**Starch polysaccharides (g/100 g)**						
Resistant starch	102	10.1–22.1	16.4 ± 0.2	***	0.31	51–111
Non-resistant starch	102	27.1–48.3	39.6 ± 0.4	***	0.37	
Total starch polysaccharides	102	44.7–68.2	56.0 ± 0.4	**	0.22	
Total prebiotic carbohydrates	102	11.4–23.2	17.5 ± 0.2			57–116

^a^%RDA is based on a recommended daily allowance of 7 g/day for sugar alcohol^40^, 7 g/day for raffinose-family oligosaccharides^41^, and 20 g/day for resistant starch and total prebiotic carbohydrates^42^. A genotype is noted as significant at **P < 0.05 and ***P < 0.01. H^2^ broad-sense heritability estimate. Total prebiotic carbohydrates include resistant starch, raffinose-family oligosaccharides, and sugar alcohols. N: number of samples.

**Figure 2:**
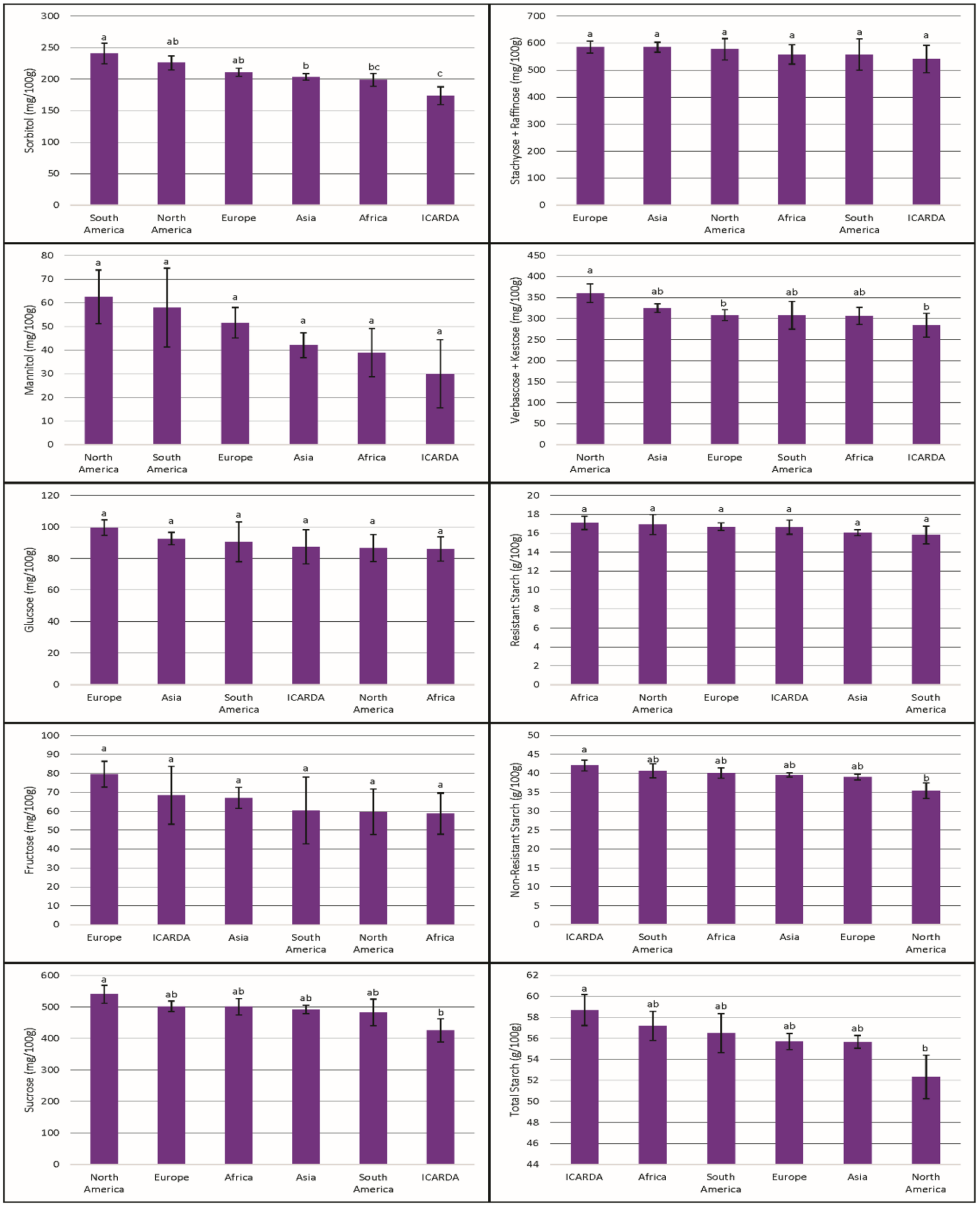
Comparison of carbohydrate concentrations by continent of origin. Bars separated by different letters have significantly different means (p < 0.05). Bars labeled as ICARDA originated as part of the ICARDA breeding program.

Significant differences in carbohydrate concentrations by continent of origin were evident for sor, suc, ver + kes, NRS, and TS (Fig. 2). SA concentrations were highest in accessions from South America (sor) and North America (man) and lowest in the ICARDA accessions. Simple sugar concentrations were highest in accessions from Europe (glu, fru) and North America (suc) and lowest in accessions from Africa (glu, fru) and ICARDA (suc). RFO concentrations were highest in accessions from Europe (sta + raf) and North America (ver + kes) and lowest in accessions from ICARDA. Finally, starch concentrations were highest in accessions from Africa (RS) and ICARDA (NRS, TS) and lowest in accessions from South America (RS) and North America (NRS, TS).

**Figure 3:**
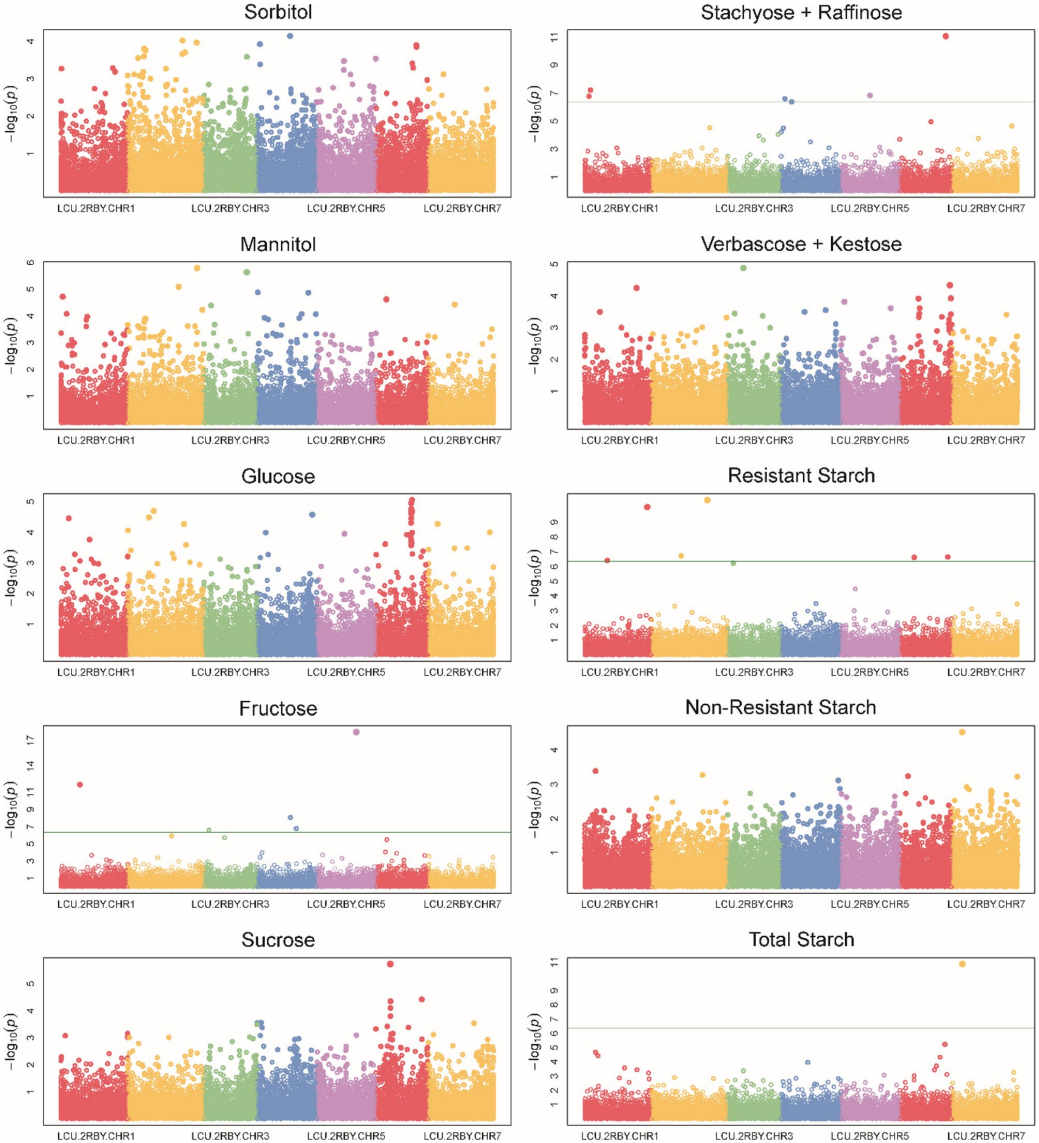
Genome-wide association study Manhattan plots from GAPIT. The green line represents the Bonferonni significance threshold (p < 0.01/22,222).

Significant single nucleotide polymorphisms (SNPs) were identified for fru, sta + raf, RS, and TS (Fig. 3, Table 3). Significant SNPs tended not to be in linkage disequilibrium with adjacent SNPs, likely due to the low coverage of GBS data and the large genome size. Three SNPs were significantly associated with man (chromosomes 2–4), with one (CHR2_558954064) identified by both software programs employed (GAPIT and GEMMA) and having a minor allele frequency (MAF) of 5.9%. One SNP was significantly associated with glu (chromosome 6). Ten SNPs were significantly associated with fru (chromosomes 1–5), two of which (CHR1_153779147, CHR5_316719059) were identified by both software programs with MAFs of 7.3 and 5.2%, respectively. One SNP was significantly associated with suc (chromosome 6) and was identified by both software programs with an MAF of 5.2%. Twenty-two SNPs were significantly associated with sta + raf (chromosomes 1, 4–7), with one (CHR6_371563912) identified by both software programs with an MAF of 9.8%. Ten SNPs were significantly associated with RS (chromosomes 1–3, 6–7), and one was significantly associated with TS (chromosome 7). Linkage blocks containing significant SNPs largely exceeded 100 kb and contained genes too numerous to include here. Genes within 100 kb flanking regions can be accessed in Supplemental Table 1.

**Table 3:** Significant SNPs identified using GAPIT and GEMMA software.

Carbohydrate	Significant SNP	p-value (GAPIT)	p-wald^a^ (GEMMA)	MAF (%)
Mannitol	CHR2_558954064	1.6E−06	7.4E−07	5.9
	CHR3_346516487	NS	1.3E−06	6.6
	CHR4_179223602*	NS	6.0E−08	8.0
Glucose	CHR6_290592280	NS	2.2E−06	14.7
Fructose	CHR1_153779145	NS	7.2E−07	7.3
	CHR1_153779147	1.3E−12	7.2E−07	7.3
	CHR1_537293922*	NS	7.4E−08	7.0
	CHR1_537449765	NS	7.4E−08	7.0
	CHR2_352993379	1.1E−06	NS	8.4
	CHR3_167167171	1.8E−06	NS	19.2
	CHR3_39693339	2.5E−07	NS	7.7
	CHR4_268011619*	8.1E−09	NS	16.8
	CHR4_316841184	1.6E−07	NS	5.2
	CHR5_316719059*	1.0E−18	4.3E−07	5.2
Sucrose	*CHR6_116880302*	1.8E−06	9.0E−07	5.2
Stachyose + raffinose	CHR1_35234757*	1.7E−07	NS	9.8
	CHR1_46070961*	6.0E−08	NS	7.0
	CHR1_143888359*	NS	4.1E−08	5.6
	CHR4_32265499	2.5E−07	NS	8.4
	CHR4_87430341	4.2E−07	NS	6.6
	CHR5_235283678	1.5E−07	NS	12.6
	CHR6_116870957	NS	4.2E−08	5.2
	*CHR6_116880302*	NS	1.6E−07	5.2
	CHR6_117946950	NS	3.8E−07	5.6
	CHR6_121916516	NS	7.0E−07	6.3
	CHR6_126221589	NS	2.0E−06	6.3
	CHR6_126869085	NS	2.0E−06	6.3
	CHR6_128918912	NS	7.0E−07	6.3
	CHR6_128918961	NS	7.0E−07	6.3
	CHR6_130768080	NS	7.0E−07	6.3
	CHR6_137205602	NS	1.1E−06	5.9
	CHR6_137205644	NS	1.1E−06	5.9
	CHR6_137214200	NS	1.9E−06	5.9
	CHR6_137214204	NS	1.9E−06	5.9
	CHR6_371563912	8.5E−12	2.6E−08	9.8
	CHR7_371621305	NS	3.6E−07	5.9
	CHR7_371621330	NS	3.6E−07	5.9
Resistant starch	CHR1_181806369*	3.8E−07	NS	6.9
	CHR1_505079023	9.0E−11	NS	8.8
	CHR2_137384845*	NS	1.0E−06	22.1
	CHR2_137480326	NS	1.1E−07	22.1
	CHR2_137480370	NS	1.1E−07	22.1
	CHR2_239215652	1.8E−07	NS	8.3
	CHR2_451413537	3.1E−11	NS	13.7
	CHR3_45534258*	6.0E−07	NS	27.0
	CHR6_116906535	2.3E−07	NS	5.2
	CHR6_387488515	2.2E−07	NS	6.9
Total starch	CHR7_84111711	1.4E−11	3.2E−07	4.9

*Located within a gene. Italicized SNP (*CHR6_116880302)* is associated with both suc and sta + raf. NS = not identified as significant by the software.

^a^GEMMA p-wald values were from the Wald test.

## Discussion

This study estimated the concentrations of 10 different carbohydrates in a lentil mapping population to understand underlying genetic mechanisms. To our knowledge, it is the first publication to identify associated SNPs and candidate genes for lentil prebiotic carbohydrates via GWAS. Furthermore, it stands as one of the few GWAS for lentils irrespective of the trait. The findings are essential for developing markers for molecular-assisted breeding approaches for nutritional and climate-change resilience breeding objectives in lentils. Prebiotic carbohydrates are important traits relevant both to human health and crop climate-change resilience. Specifically, a healthy gastrointestinal microbiome is sustained mainly by consuming prebiotic carbohydrates in the human diet, which promote the growth of beneficial microorganisms, such as *Lactobacilli* and *Bifidobacteria*^22^. A healthy microbiome has been associated with numerous health benefits, including increased mineral absorption and reduced risk of colon cancer, diabetes, irritable bowel disease, and others^9^. In addition, these carbohydrates play an essential role in increasing the plant's abiotic stress tolerance, being associated with tolerance to salinity, heat, cold, and freezing stresses^12–15^.

Low-molecular-weight carbohydrate concentrations were generally consistent with values found in the literature for lentils; however, mean concentrations of sor, suc, sta + raf, and ver + kes were on the low end of normal^5,23,24^. Typical lentil SA concentration ranges are 1000–2000 mg/100 g (sor) and 50–300 mg/100 g (man); values measured here are notably lower for sor (113–328 mg/100 g) and similar for man (2–357 mg/100 g). Typical simple sugar concentration ranges are 20–300 mg/100 g glu, 0.2–50 mg/100 g fru, and 1000–2500 mg/100 g suc; values measured here are similar for glu (36–315 mg/100 g), higher for fru (7–325 mg/100 g), and lower for suc (208–1010 mg/100 g). Typical RFO concentrations are 1500–5000 mg/100 g sta + raf and 500–2500 mg/100 g ver + kes; values measured here are both notably lower at 344–1748 mg/100 g sta + raf and 164–647 mg/100 g ver + kes. Total starch concentrations were consistent with the literature^5,23^; however, RS concentrations were higher than expected based on literature values, at 10–22 g/100 g compared to 5–10 g/100 g. This also corresponded to lower NRS values than expected. Overall, significant variation was evident within this population grown under greenhouse conditions. Larger variation in concentrations would be expected in field trials in addition to genotype × environment effects.

Heritability estimates showed cautious potential for breeding for these traits. Sugar alcohols' broad-sense heritability estimates are not commonly calculated in grain crops. Sorbitol heritability estimate in peach was reported as 0.7–0.8^25^, which is higher than noted for lentil in the present study (0.34). Estimates for simple sugar and RFO heritabilities are consistent with other literature on pulse crops. H^2^ values for glucose and sucrose (0.20 and 0.34) are compatible with other pulse crops, ranging from 0.2–0.4 and 0.2–0.5, respectively^26,27^. The H^2^ value for fructose is high compared to 0.05–0.07 in chickpea^26^. The H^2^ value for stachyose + raffinose of 0.41 is comparable to heritabilities of 0.2–0.5 in common bean and desi and kabuli chickpea^26,27^. Resistant starch (H^2^ = 0.31) is a novel phenotype for which heritability estimates are limited; however, total starch heritability of 0.3–0.4 has been reported in barley^28^, which is slightly higher than the value of 0.22 for lentil in the present study. This study indicates low to medium heritability estimates for lentil prebiotic carbohydrates, suggesting that the environment may play a more significant role than genotype in determining these concentrations; this may challenge breeding for these traits. However, this is the first study to measure heritability in these traits for lentils and was performed in a controlled greenhouse environment, so it is too early to make any definitive statements for or against breeding prospects. Field trials with multiple locations will be vital toward estimating heritability more accurately and determining genotype × environment effects. In addition, increasing the lentil population size to encompass broader genetic diversity will potentially increase heritability estimates.

Based on %RDA values, there is significant potential within the *Lens culinaris* species for selecting lentil lines of high or low prebiotic carbohydrate content. Our results also suggest the potential for incorporating prebiotic carbohydrates as a nutritional trait in breeding programs. From a dietary perspective, specific lentil accessions may be selected based on their prebiotic concentration, potentially providing up to 100% of the RDA. Human populations with obesity would benefit from varieties with increased prebiotic carbohydrate levels; these varieties may also increase climate resilience for global food security. For populations where specific prebiotics in lentil may cause undesirable side effects, including bloating, flatulence, indigestion, need lentil cultivars with lower total prebiotic concentrations, or particular carbohydrates could be targeted, such as RFOs, which are the carbohydrate family primarily implicated in indigestion^29^. Target concentrations may vary depending on the desired outcome and population; nevertheless, RS, which makes up most prebiotic content in lentils, may prioritize the most significant trait of interest. Whereas non-resistant starch is digested and absorbed in the upper digestive tract, RS is not broken down by digestive enzymes and consequently enters the colon, fermented by microorganisms^30^.

Prebiotic carbohydrate concentrations vary by growing location^24^. The present study showed that some prebiotic carbohydrate concentrations also vary by continent of origin, although this difference is not significant in most cases. This result can be interpreted with contrasting ramifications. In the cases where little difference is detected (man, sta + raf, and RS), this may suggest that the trait is highly conserved. If so, the lentil plant must tightly regulate these concentrations to produce viable seed; manipulating these concentrations through breeding would then be challenging and, if successful, may have a detrimental effect on the plant and agronomic traits, including yield.

In contrast, where concentrations differ by continent of origin (sor, ver + kes) may suggest that prebiotic carbohydrate concentrations have been under selective pressure in the lentil's evolutionary development^31^. During lentil’s introduction to new regions, differences in climate would have been a prominent source of pressure driving variation alongside historical agronomic breeding. If prebiotic carbohydrate concentrations played a role in these historical adaptations, exploring their potential in developing varieties resilient under various environmental conditions is warranted. Namely, the warmer, dryer climates feared to result from climate change. More studies, including a larger population and multiple field trials, are needed to support these hypotheses with heritability.

GWAS has been successfully used in other crops to identify significant SNPs and candidate genes for simple sugars and RFOs^32,33^. Few GWAS on lentil have been reported in the literature, likely due to the lack of genetic resources. The development of genetic resources for lentil and other legumes has lagged behind other crops, such as maize and sorghum. For example, the lentil genome remains unpublished, in part due to its size and repetitive nature. In addition, the quality of the genome available through the University of Saskatchewan was relatively poor until the recent release of version 2.0, which incorporated multiple sequencing platforms as well as long and short reads (presentation and communication with Kirsten Bett of University of Saskatchewan at North American Pulse Improvement Association, Fargo, ND, Nov 6–8, 2019).

This GWAS on lentil prebiotic carbohydrates uncovered several significantly associated SNPs. SNP markers were identified for the prebiotic carbohydrates man, sta + raf, RS, and the non-prebiotic carbohydrates glu, fru, suc, and TS. Due to the ubiquity of SNPs in the genome, they are convenient markers for GWAS. Though a significant SNP is often not the causative mutation, it may be in linkage with the causative mutation. Genes within 100 kb of each significant SNP are shown in Supplemental Information. A number of significant SNPs were identified within genes. For example, CHR1_143888359 was located within Lcu.2RBY.1g019390, homologous to a galacturonosyltransferase in *Arabidopsis thaliana*. Generally, this gene class is known for the synthesis of pectin in cell walls^34^; however, the transfer of galactose is the primary step in RFO synthesis carried out by galactosyltransferases^35^. Thus, this discovery offers a potential gene target for altering RFO concentration in lentil.

## Conclusion

Lentil prebiotic carbohydrates play a vital role in plant physiology and should be further explored as a means of breeding lentil varieties for changing climates. Additionally, prebiotic carbohydrates are important for human health, specifically for their role in regulating and modulating the gut microbiome. Thus, increased consumption of lentil and other pulse crops could have a beneficial effect on many people's health. Future studies should validate identified candidate genes to verify their function and uncover causative mutations. Once confirmed, markers can be confidently developed for molecular-assisted breeding for prebiotic carbohydrates. Markers, such as microsatellites, could be used in molecular-assisted breeding approaches to incorporate the desired alleles and then recover the elite cultivar genotype through backcrossing aided by markers scattered across the genome^36^.

## Materials and methods

### Materials

Standards, chemicals, and high-purity solvents used for prebiotic carbohydrate analysis were purchased from Sigma Aldrich Co. (St. Louis, MO), Fisher Scientific (Waltham, MA), VWR International (Radnor, PA), and Tokyo Chemical Industry (Portland, OR) and used without further purification. Water, distilled, and deionized (ddH_2_O) to the resistance of ≥ 18.2 MΩ × cm (PURELAB flex 2 system, ELGA LabWater North America, Woodridge, IL) was used for sample and reagent preparation.

### Greenhouse

Two lentil mapping populations were obtained from the International Centre for Agricultural Research in the Dry Areas (ICARDA), Rabat-Institute, Rabat, Morocco. The heat tolerance population (150 accessions) and the global mapping population (128 accessions) were grown in a completely randomized design with two replicates (n = 558) at the Clemson University Greenhouse Complex, Clemson, SC, USA (Table 1). The soil in each pot was saturated with ddH_2_O and allowed to drain overnight. At seeding, pots were at 80% pot capacity. Greenhouse conditions were day and night temperatures of 22/20 °C. Photosynthetically active radiation levels were 300 µmol/m^2^/s using a 16-h photoperiod and 50–60% relative humidity. All pots were watered to approximately 70% of free-draining moisture content every day, and 250 mL of the nutrient solution were added to all pots every 2 weeks, as per standard procedures for lentils at the Clemson University Pulse Quality and Nutrition program. Nutrient concentrations of the all-purpose 20-20-20 fertilizer solution (Plant Products Co. Ltd., Brampton, ON, Canada) were 20% total N, 20% total P, 20% soluble K, 0.02% B, 0.05% chelated Cu, 0.1% chelated Fe, 0.05% Mo, 0.05% Zn, and 1% EDTA. All plants were hand-harvested at physiological maturity, air-dried (40 °C), and hand-threshed. The total seed weight per pot was recorded, and the seeds were stored at − 40 °C until analysis.

### Low molecular weight carbohydrate or prebiotic carbohydrate analysis

Lentil seeds were ground (Blade Coffee Grinder, KitchenAid, St. Joseph, MI, USA) and sieved to 0.5-mm particle size. Carbohydrates were extracted following Muir et al.^37^ with modification. Each flour sample was weighed (150 mg) into a centrifugal polypropylene tube (VWR International, Radnor, PA, USA). After adding 10 mL of water, each tube was mixed on a vortex mixer and placed in a water bath for 1 h at 80 ℃. Tubes were then centrifuged at 3000*g* for 10 min. The supernatant was filtered through a 13 mm × 0.45 μm nylon syringe filter (Thermo Fisher Scientific, MA, USA) into an HPLC vial for analysis.

Low molecular weight carbohydrate analysis was performed following Feinberg et al.^38^ on a Dionex ICS-5000^+^ system (Thermo Scientific, Waltham, MA, USA) equipped with a pulsed amperometric detector (PAD) with a working gold electrode and a silver-silver chloride reference electrode. The separation was achieved using a Dionex CarboPac PA1 analytical column (250 × 4 mm) in series with a Dionex CarboPac PA1 guard column (50 × 4 mm). Pure standards were used to identify peaks, generate calibration curves, and monitor detector sensitivity. A lentil lab reference sample was used to monitor extraction consistency. Concentrations were quantified within a linear range of 0.1–500 ppm with a minimum detection limit of 0.1 ppm. Concentrations in samples were calculated following X = (C × V)/*m*, where X is the moisture-corrected analyte concentration in the sample, C is the concentration in the filtrate, V is the sample volume, and *m* is the mass of the dry lentil flour.

### Starch analysis

Resistant, non-resistant, and total starch were measured using the AOAC approved Megazyme resistant starch assay method^39^. Each sample was weighed (100 mg) into a centrifugal polypropylene tube. Enzyme solution was added (2 mL), which contained amyloglucosidase (3 U/mL) and αּ-amylase (10 mg/mL) in sodium maleate buffer (100 mM, pH 6.0). Tubes were incubated with constant circular shaking (200 strokes/min) for 16 h at 37 ℃. Ethanol (4 mL; 99%) was added, followed by vortex mixing centrifugation (1500*g* for 10 min) and decanting into 100-mL volumetric flasks. Two additional washings of the sample were performed, adding 2 mL of ethanol (50%) and vortex mixing to suspend the pellet, followed by an additional 6 mL of ethanol (50%), vortex mixing, centrifugation, and decanting. Pooled non-resistant starch washings were brought to 100 mL volume with water. Pellets containing resistant starch were dissolved in 2 mL of 2 M KOH with a magnetic stir bar for 20 min in an ice water bath. Sodium acetate buffer (8 mL, 1.2 M, pH 3.8) was added, followed immediately by 0.1 mL of amyloglucosidase (AMG; 3300 U/mL). Samples were incubated at 50 ℃ in a water bath for 30 min. Tubes were then centrifuged (1500*g* for 10 min). Resistant starch and non-resistant starch fractions were quantified via spectrophotometry as follows. Starch solution (0.1 mL) and glucose oxidase/peroxidase (GOPOD) reagent (3 mL) were added to a glass tube and incubated for 20 min at 50 ℃. A glucose standard (1 mg/mL in 0.2% benzoic acid) was included in each batch. Absorbance was measured at 510 nm against a reagent blank. Non-resistant starch was calculated by the formula NRS (g/100 g sample) = ΔE × F/W × 90, where ΔE is the absorbance of the sample, F is the absorbance to microgram conversion factor (100/absorbance of glucose standard), W is the sample dry weight, and 90 includes adjustments for volume, unit conversions, and free to anhydrous glucose. Resistant starch was calculated by a similar formula: RS (g/100 g sample) = ΔE × F/W × 9.27, where 9.27 includes adjustments for volume, unit conversions, and free to anhydrous glucose. Total starch was calculated as TS = RS + NRS.

### Statistical analysis

Carbohydrate concentration means, standard errors, and ranges were averaged across replications for each accession. Carbohydrate distributions were displayed as histograms, and normal curves were fit to the histograms to determine how closely the values followed a normal distribution. To compare each carbohydrate concentration among a continent of origin, a statistical model was developed with the mean concentration of each carbohydrate as the response variable and continent as a fixed effect. The model was estimated using standard least squares. ANOVA was used to determine if the continent effect was significant. Fisher's Protected Least Significant Difference Test was used to compare mean concentrations by continent of origin for each carbohydrate. P-value < 0.05 was considered evidence of statistical significance. To estimate broad-sense heritability (H^2^), a statistical model was developed with the mean concentration of each carbohydrate as the response variable and genotype as a random effect. The model was estimated using the restricted maximum likelihood (REML) method. H^2^ was identified as the proportion of variance due to genotype. Percent recommended daily allowances (%RDA) were calculated for total SA, total RFO, and RS, and total prebiotic carbohydrate concentrations based on 7 g/day for sugar alcohols, 7 g/day for RFOs, 20 g/day for RS, and 20 g/day for total prebiotic content^40–42^. All calculations were performed using JMP 14.0.0.

### Genome-wide association study

Previously sequenced genotyping-by-sequencing (GBS) data were used for genome-wide association analysis^21^. The TASSEL-GBS pipeline^43^ with default parameters was used for aligning reads to the reference genome (Lens culinaris v2.0) and for single nucleotide polymorphism (SNP) calling. Beagle 5.0 with default settings was used for imputation^44^. VCFTools was used for filtering the VCF file to include only the 143 lentil lines included in the study (102 for starch) and to exclude sites with less than 5% minor allele frequency (MAF) and more than 20% missing data, leaving 22,222 high-quality SNPs for analysis^45^. Association analyses were conducted with two software programs and models: the Genome Association and Prediction Integrated Tool (GAPIT) in R using the FarmCPU model^46^ and the Genome-wide Efficient Mixed Model Association Algorithm (GEMMA) using a linear mixed model for univariate analyses^47^. Least square means from the JMP analysis were used. The population structure was estimated with the VanRaden kinship matrix algorithm in GAPIT. PLINK^48^ was used to calculate linkage disequilibrium decay around significant SNPs to determine linkage blocks and identify candidate genes from a GFF3 file.

## Supplementary Information

Supplementary Information.
